# Effect of Adhesive Type and Surface Preparation on the Debonding Behavior of Glass and Carbon Fiber Reinforced Epoxy Adhesive Joints

**DOI:** 10.3390/ma19081561

**Published:** 2026-04-14

**Authors:** Paula Vigón, Antonio Argüelles, Miguel Lozano, Jaime Viña

**Affiliations:** 1 Department of Construction and Manufacturing Engineering, Edificio Departamental Oeste, n 7, University of Oviedo, 33203 Gijón, Spain; vigonpaula@uniovi.es (P.V.); antonio@uniovi.es (A.A.); 2 School of Engineering and Technology (ESIT), Universidad Internacional de La Rioja (UNIR), 26006 Logroño, Spain; miguel.lozano@unir.net; 3 Department of Materials Science and Metallurgical Engineering, Edificio Departamental Este, n3, University of Oviedo, 33203 Gijón, Spain

**Keywords:** debonding, surface preparation, CFRP, Mode I, glass fiber, carbon fiber, fracture

## Abstract

In this work, the debonding behavior under quasi-static Mode I fracture loading of adhesive joints made on two types of composite materials with the same epoxy matrix and unidirectional carbon or glass fiber reinforcement was analyzed. Standard DCB tests were used to quantify the influence of adhesive type and substrate surface preparation on interlaminar fracture toughness. For the fabrication of the joints under study, three commercial structural adhesives from different manufacturers were selected, two epoxy-based and one acrylic-based. Substrate surface preparation was carried out using three different procedures: manual abrasion, sanding with P220 Al_2_O_3_ sandpaper, grit blasting with Al_2_O_3_, and peel ply PA80 polyamide fabric. The experimental results revealed the same trend for both epoxy-based adhesives: sanding provided the best results, regardless of the substrate used. Surface preparation by grit blasting proved highly sensitive to the applied parameters, generally yielding poorer results than manual sanding. Surface preparation using PA80 peel ply fabric may be a viable option. However, its main drawback is that it must be incorporated during composite manufacturing. The results demonstrate that fracture performance is governed by the interaction between adhesive chemistry and surface morphology rather than by surface roughness alone.

## 1. Introduction

Fiber-reinforced polymer composites have become essential structural materials in many engineering applications due to their high specific stiffness and strength, corrosion resistance, and design flexibility. Among them, carbon fiber reinforced polymers (CFRP) and glass fiber reinforced polymers (GFRP) represent two of the most widely used solutions, offering different balances between mechanical performance, cost, and durability [1]. CFRP laminates are typically selected for high-performance lightweight structures [2], while GFRP composites provide a more economical alternative for components where moderate stiffness and strength are required. Consequently, both materials are frequently employed in bonded structural assemblies, in which the integrity of the adhesive joint plays a decisive role in the global mechanical response [3].

Despite their excellent mechanical performance, laminated fiber reinforced composites are susceptible to interlaminar damage mechanisms [4,5,6], among which debonding [7] is one of the most critical damage mechanisms affecting the structural integrity of adhesively bonded composite laminates [8,9], as reported in several experimental studies [10,11]. The initiation and propagation of debonding can significantly reduce the load-bearing capacity and stiffness of structural components, particularly when adhesive bonding is used to assemble composite parts.

Surface preparation of composite substrates is widely recognized as a critical factor governing the performance of adhesively bonded joints [12,13]. The effectiveness of the bonding process strongly depends on the ability to modify the physical and chemical characteristics [14] of the interface by promoting adequate surface roughness, removing weak boundary layers and contaminants, and increasing surface energy, thereby improving both mechanical interlocking and physicochemical adhesion mechanisms. In fiber-reinforced composites, these aspects are particularly relevant due to the heterogeneous nature of the material and the sensitivity of the matrix–fiber interface to damage initiation.

Consequently, a wide range of preparation techniques, including mechanical abrasion [15], grit blasting [16], peel ply application, and chemical treatments, have been investigated to optimize the interfacial conditions prior to bonding [17]. These treatments may generate substantially different surface morphologies and interfacial properties [18], as well as adhesive thickness [19,20], which can directly influence crack initiation, crack propagation, and overall debonding resistance of bonded composite structures [21]. For this reason, considerable research effort has been devoted to identifying the parameters governing the mechanical response of adhesively bonded joints [22,23], such as the mechanical and physical properties of both adherends and adhesives [24], joint geometry, and loading conditions.

In particular, the influence of adhesive type has attracted increasing attention [25], since different adhesive chemistries may lead to distinct stiffness levels [26], energy dissipation mechanisms, and fracture behaviors [27,28]. Structural epoxy adhesives are commonly used in composite bonding due to their high stiffness and strength [29]. In contrast, acrylic-based adhesives can provide improved toughness and damage tolerance under certain loading conditions [30,31].

Further research has focused on the effects of testing methodologies [32,33], loading regimes [34,35], and environmental degradation [36,37,38] on debonding initiation and growth in bonded composite joints [39]. Other studies have investigated the incorporation of interlayers or reinforcing elements to improve adhesion performance [40]. Furthermore, the durability of adhesively bonded composite joints under environmental degradation processes, such as moisture exposure [41], saline environments [42,43], and hygrothermal effects [44], has also received considerable attention. Parallel efforts have been devoted to the development of predictive tools for the design and validation of bonded composite structures [45].

However, the combined influence of adhesive chemistry and substrate surface preparation on the debonding resistance of bonded joints in different fiber reinforced composite laminates has not yet been fully clarified [46].

In this context, the present work aims to evaluate the influence of different surface preparation techniques on the debonding behavior of adhesively bonded joints manufactured from composite laminates with the same epoxy matrix and reinforced with either unidirectional carbon or glass fibers. Three commercial structural adhesives were considered: two epoxy-based systems compatible with the composite matrix and one acrylic-based adhesive. The resistance to debonding was characterized through the Mode I energy release rate obtained from standard Double Cantilever Beam tests.

## 2. Materials

### 2.1. Base Materials

The composite materials used in this work as the baseline substrates were of two different types. Both employed the same epoxy matrix (MTC510, SHD Composites Ltd., Sleaford, UK) and were supplied as prepregs by the same manufacturer. One consisted of unidirectional high-strength carbon fibres (HS grade), designated MTC510-UD300-HS-33%RW. The other consist of unidirectional E-glass fibers (stitched), with the commercial designation MTC510-StitchedUD300-Eglass-37%RW.

The mechanical properties of both laminates are presented in Table 1, together with the coefficient of variation (CV) of the measurements. These were obtained from experimental tests carried out by the authors.

The laminates were produced by vacuum bag molding following the curing cycle recommended by the manufacturer (ramp to 120 °C and hold for 6 h). Once cured, the panels were cut into rectangular plates measuring 225 mm × 20 mm × 2.75 mm.

### 2.2. Adhesives

Three commercial adhesives were used: two epoxy-based (Loctite^®^ EA 9461™ and Araldite^®^ 2015) and one acrylic-based (3M™ DP8010NS), all manufactured by their respective companies (Henkel AG & Co. KGaA, Düsseldorf, Germany; Huntsman Advanced Materials, Basel, Switzerland; and 3M Company, St. Paul, MN, USA) to bond each of the parts that would form the final laminate, whose surfaces had been previously treated.

Table 2 summarizes the basic technical characteristics of the three adhesives employed.

Each adhesive was cured following the cycle recommended by its respective manufacturer.

## 3. Experimental Work

The following section describes the main aspects of the experimental program carried out.

### 3.1. Specimen Preparation

The laminates were manufactured by vacuum bag molding (vacuum-assisted molding), an out-of-autoclave processing route widely used in industrial composite manufacturing. The reinforcing fibers were laid up in a unidirectional 0° orientation. The substrates were assembled by secondary bonding—i.e., two pre-cured laminates were adhesively joined by curing an adhesive layer between them. A 12 μm PTFE (Teflon) film was inserted at one end of the bondline to act as a debonding starter.

The resulting laminates were machined using a diamond saw to obtain the specimens used in the tests, with a nominal width of 20 mm and a total length of 150 mm, and an initial crack length of 50 mm from the loading line to the crack front. The total thickness of each specimen was 4.3 ± 0.1 mm for the carbon-based composite (MTC510-UD300) and 6.32 ± 0.2 mm for the glass-based composite (MTC510-StitchedUD300-Eglass). Some specimens without adhesive were also manufactured in order to evaluate the differences in behavior with respect to the baseline material.

### 3.2. Selection of the Methodology for Surface Preparation

A preliminary step prior to the experimental program was the definition of the substrate surface preparation processes prior to bonding. Four procedures were analyzed: manual sanding, grit blasting, chemical etching, and polyamide peel ply PA80. Sanding was performed using P220 Al_2_O_3_ sandpaper (Würth, Künzelsau, Germany). Grit blasting was carried out using a CAT210 sandblasting cabinet (MetalWorks brand, ASLAK Machines & Tools, Sant Quirze del Vallès, Spain), employing two types of abrasives, glass microbeads and aluminum oxide (Al_2_O_3_, corundum), with a projection time of 5 s. Chemical etching was conducted using two different procedures: the first consisted of a mixture of HNO_3_ (65%) and HCl (37%) in equal proportions, with an immersion time of 5 h; the second involved immersion in HNO_3_ (65%) at 80 °C for 3 min. In both cases, the specimens were rinsed with warm water and subsequently dried in an oven at 50 °C for 2 h.

Finally, substrates were surface prepared using polyamide peel ply PA80 during their manufacturing process. It should be noted that the main limitation of this technique is that it must be applied during the composite manufacturing stage, which significantly restricts its applicability in repair scenarios.

In all cases, after surface treatment, the substrates were cleaned with acetone and dried using compressed air prior to bonding with the selected adhesives. Surface roughness was measured both before and after the surface treatment.

The specimen edges were painted and marked from the initial crack tip up to 50 mm to facilitate crack length measurement. Markings were applied every 1 mm within the first 10 mm and every 5 mm thereafter. Crack growth was monitored during testing using a PULNiX TM-7CN camera equipped with a 50× magnification lens.

The adhesive thickness was measured on three specimens for each adhesive using a ZEISS stereomicroscope (Stemi 508) and an Axiocam 208 Color camera (both from Carl Zeiss Microscopy GmbH, Jena, Germany). Measurements were carried out at magnifications ranging from 3.2× to 5×. The average thickness values were 0.278 mm for Loctite^®^ EA 9461™, 0.215 mm for 3M™ DP8010NS, and 0.255 mm for Araldite^®^ 2015.

### 3.3. Experimental Procedure

**Figure 1:**
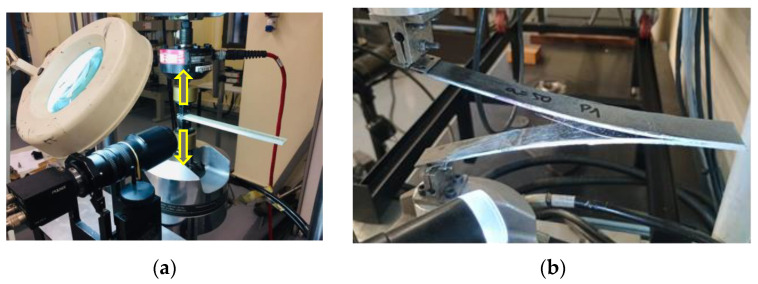
DCB experimental setup. (**a**) An optical monitoring system used to track crack growth during the test, with the applied loading direction indicated by arrows. (**b**) DCB specimen mounted in the testing frame, showing the loading hinges.

In all cases studied, five DCB specimens were tested for each combination of substrate material (two types), surface preparation (four conditions), and adhesive (three types), resulting in a total of 120 specimens. Mode I interlaminar fracture tests (Double Cantilever Beam, DCB) were conducted in accordance with ASTM D5528M-21 [49]. Figure 1 illustrates the test configuration used in the experimental setup.

All specimens were tested using an MTS 810 servo-hydraulic testing machine (MTS Systems Corporation, Eden Prairie, MN, USA) equipped with a 5 kN load cell, at a constant crosshead speed of 2 mm/min and under ambient temperature. Crack propagation was monitored using a high-resolution camera.

### 3.4. Mode I Fracture

To determine the Mode I energy release rate, G_IC_, ASTM D5528-13 was followed. Among the formulations proposed by this standard, the Modified Beam Theory was employed, according to the following expression:

G_IC_ = 3Pδ/(2b(a + ∣Δ∣)) (1)

where b is the specimen width, P is the applied load, δ is the displacement at the loading point, a is the debonding crack length, and Δ is a correction factor obtained experimentally by plotting the cube root of the compliance, $C^{\frac{1}{3}}$, as a function of the crack length, a, and performing a linear least-squares fit. The intercept of this line with the horizontal axis corresponds to the value of Δ, which accounts for crack tip rotation and deviations from ideal beam behavior. By using the effective crack length (a + Δ), the method corrects the system compliance, ensuring that the calculated G_IC_ values are not affected by differences in initial stiffness due to variations in substrate thickness or material properties.

## 4. Results

This section presents the experimental results obtained from surface characterization and Mode I fracture testing. First, the effects of the selected surface preparation techniques on substrate morphology are analyzed. Subsequently, the mechanical response under DCB loading is discussed in terms of load displacement behavior, strain energy release rate (G_IC_), and failure mechanisms.

### 4.1. Surface Characterization After Surface Preparation

The influence of the selected surface preparation techniques on substrate topography was first evaluated through roughness measurements and SEM analysis. Table 3 summarizes the main roughness parameters obtained for each treatment.

The parameters considered were as follows:

R_a_: arithmetic mean of the absolute values of the roughness profile.

R_z_: arithmetic mean of consecutive peak-to-valley distances.

R_max_: individual value of the maximum peak-to-valley height.

Based on the preliminary results obtained, the following substrate preparation methods were selected: sanding, grit blasting (specifically corundum blasting with 5 s exposure, since increasing the exposure time, although leading to higher roughness, also caused undesirable mechanical wear on the substrate surface), and Peel Ply.

The chemical etching processes were discarded because, for both exposure conditions, they were shown to generate residual contamination on the substrate.

**Figure 2:**
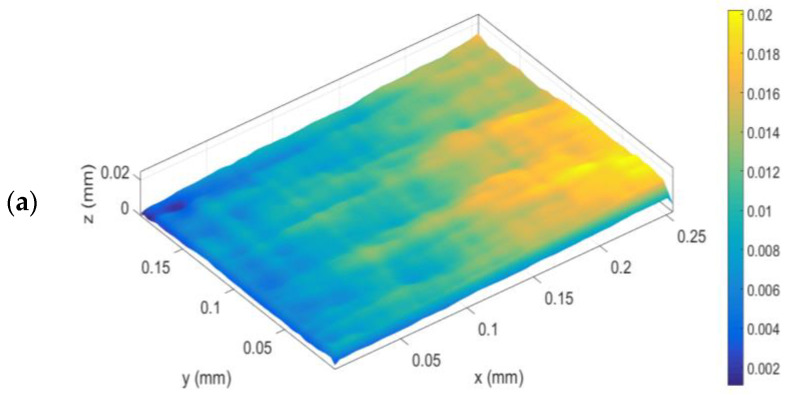

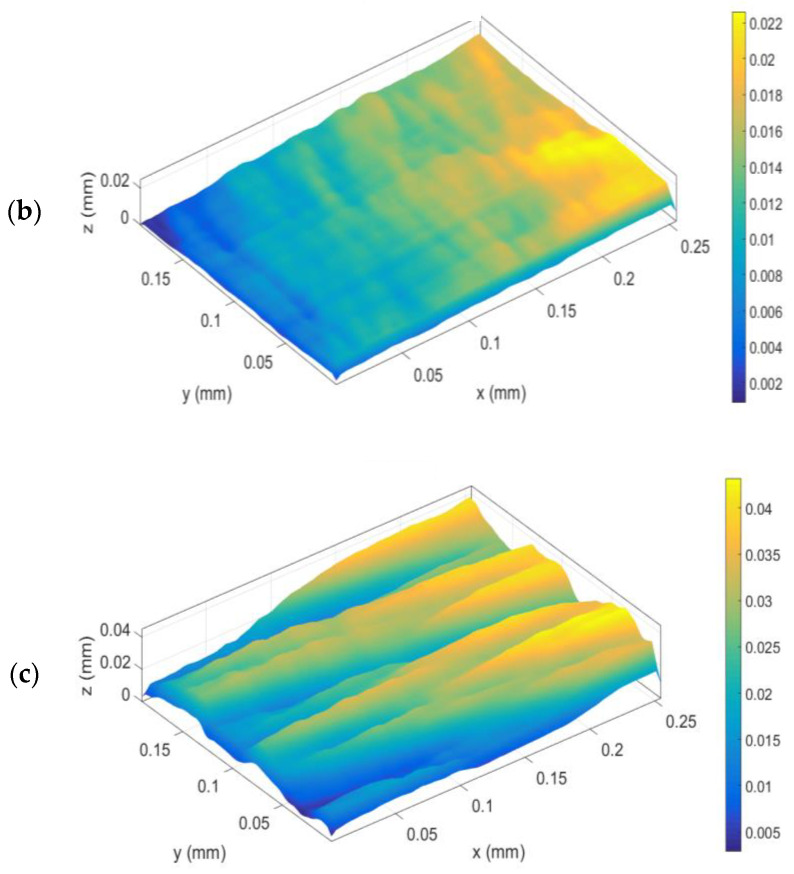
SEM 3D surface images of the selected treatments at ×50 magnification: (**a**) Al_2_O_3_ sanding; (**b**) Al_2_O_3_ blasting; (**c**) Peel Ply PA80.

Once the surface treatments were selected, an analysis of the resulting substrate surfaces was carried out by scanning electron microscopy (SEM). The roughness of the substrates with the different surface preparations was observed in 3D, obtained from a three-dimensional reconstruction under different apparent illumination conditions. Figure 2 shows the different images obtained.

The roughness values, together with the SEM images, showed higher roughness for the peel ply technique, while no significant differences were observed between sanding and grit blasting treatments, the latter exhibiting slightly higher values.

### 4.2. Mode I DCB Load Displacement

**Figure 3:**
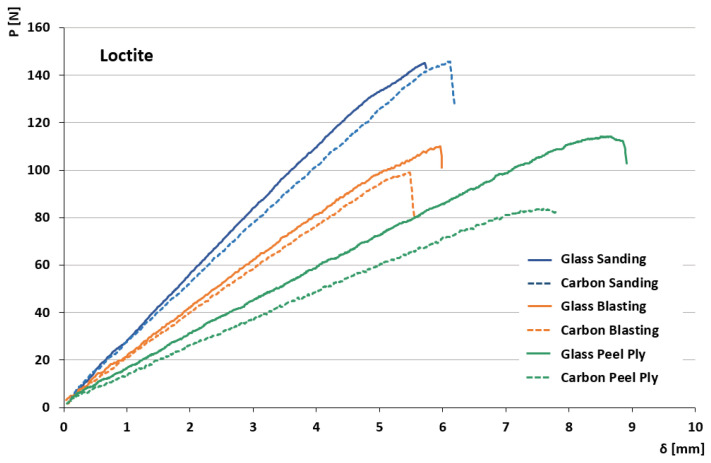
Load–displacement curves for both materials using Loctite adhesive.

Figure 3 presents the load displacement curves obtained from the Mode I fracture initiation tests for some specimens considered representative of the behavior of both substrates used in this study (carbon-based and glass-based), manufactured using Loctite as the adhesive. Similar trends were observed for the other adhesives.

It can be observed, regardless of the type of substrate used, that the sanding process produced higher delamination loads compared to grit blasting, although the differences are small, as shown in Figure 3. The use of peel ply did not improve the maximum load values for either substrate; however, higher displacement values were observed, along with noticeably lower slopes in the load–displacement curves, which indicates a reduction in specimen stiffness in the specimens manufactured with this technique.

Regarding the stiffness achieved by the adhesive joint and its relationship with the surface preparation process, it was generally observed that sanding resulted in the highest stiffness values, although close to those obtained with grit blasting. In addition, the higher stiffness of the specimens manufactured with glass substrates can be attributed to the greater thickness of the tested material.

Noticeable differences in the initial slopes can be observed among the configurations. These variations are discussed below.

The differences observed in the initial slopes of the load–displacement curves (Figure 3) are primarily attributed to variations in specimen compliance. In DCB configurations, the initial stiffness depends on the elastic modulus of the substrates, the arm thickness, and the initial crack length. Since two different substrate materials with distinct thicknesses were evaluated, variations in the initial linear response are expected.

Although all specimens were pre-cracked according to ASTM D5528-13, minor differences during the transition from the insert to a natural crack may slightly affect the early loading stage and apparent maximum load. However, fracture toughness values were calculated using the Modified Beam Theory (MBT), which accounts for compliance corrections and ensures that the reported G_IC_ values are not influenced by these initial stiffness differences.

### 4.3. Mode I Energy Release Rate G_IC_

**Figure 4:**
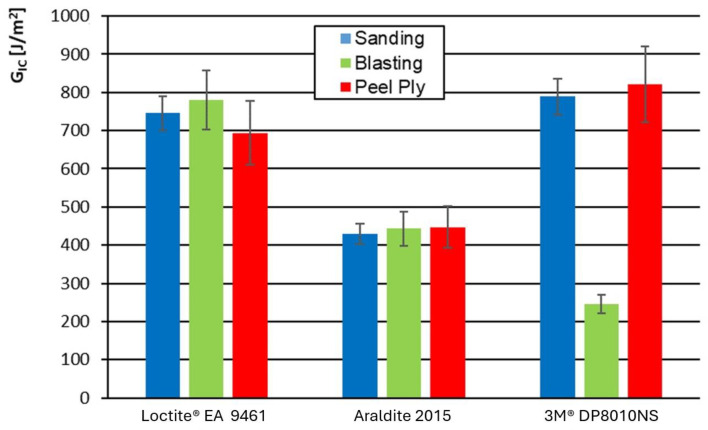
Mode I energy release rate as a function of adhesive type and surface preparation method for the carbon-based composite.

Figure 4 shows the Mode I energy release rate for each substrate surface preparation technique, for the carbon-fiber composite, and the three adhesives used.

The most homogeneous results were obtained for the Loctite adhesive, regardless of the surface preparation technique employed. Araldite exhibited the lowest Mode I fracture resistance. For the acrylic 3M adhesive, its weakest performance was noted when grit blasting was used as the surface preparation technique.

**Figure 5:**
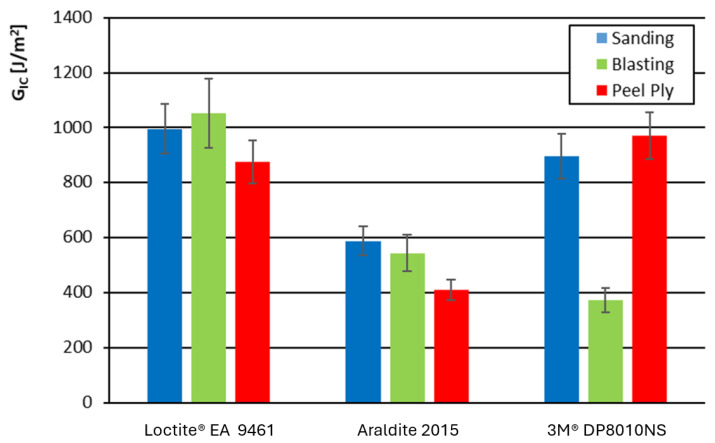
Mode I energy release rate as a function of adhesive type and surface preparation method for the glass-based composite.

Figure 5 shows the Mode I energy release rate (G_IC_) as a function of adhesive type and surface preparation method for the glass-fiber composite where the error bars represent the standard deviation of these five measurements (*n* = 5).

For the glass-fiber-reinforced substrate, the highest G_IC_ values were obtained with the Loctite adhesive, regardless of the surface preparation technique employed, whereas Araldite exhibited the lowest fracture toughness values.

Although CFRP and GFRP laminates exhibit different stiffness in the longitudinal direction (E_11_ ≈ 122 GPa for CFRP and 38.8 GPa for GFRP, approximately three times higher for CFRP, see Table 1), similar fracture trends were observed for both substrates. This suggests that interfacial and adhesive-related mechanisms played a more dominant role in governing fracture behavior than the intrinsic stiffness differences between carbon- and glass-fiber reinforcements.

The acrylic adhesive 3M™ DP8010NS showed a marked reduction in Mode I fracture toughness under grit blasting conditions, similarly to the behavior observed for the carbon-fiber-reinforced substrate.

The reduced fracture toughness observed for 3M™ DP8010NS after grit blasting can be attributed to interfacial effects rather than to the intrinsic cohesive properties of the adhesive. 

Grit blasting modifies the composite surface by partially removing the resin-rich layer and locally exposing fibers, while also increasing surface roughness and potentially embedding abrasive particles. For an acrylic-based adhesive such as DP8010NS, which relies strongly on surface chemistry and wetting behavior, these alterations may reduce interfacial compatibility and hinder effective stress transfer.

It is important to note that surface roughness alone does not govern fracture performance. Although grit blasting increases surface roughness and peel ply treatment may produce even higher roughness values, the strain energy release rate, which represents the energy required for crack propagation, is governed by the combined effect of surface morphology, chemical compatibility, and the resulting failure mechanism along the crack path. Peel ply generates a controlled, resin-rich surface that promotes cohesive failure and efficient energy dissipation, whereas grit blasting may alter surface chemistry and reduce interfacial compatibility for certain adhesives.

Therefore, an increase in roughness does not necessarily lead to improved fracture toughness; the adhesive–substrate interaction plays a dominant role in determining joint performance.

Fiber bridging was qualitatively observed in several configurations, particularly for epoxy-based adhesives. Although no quantitative measurement of the bridging length was performed, its presence is consistent with the higher fracture toughness values recorded and contributes to increased energy dissipation during crack propagation.

**Figure 6:**
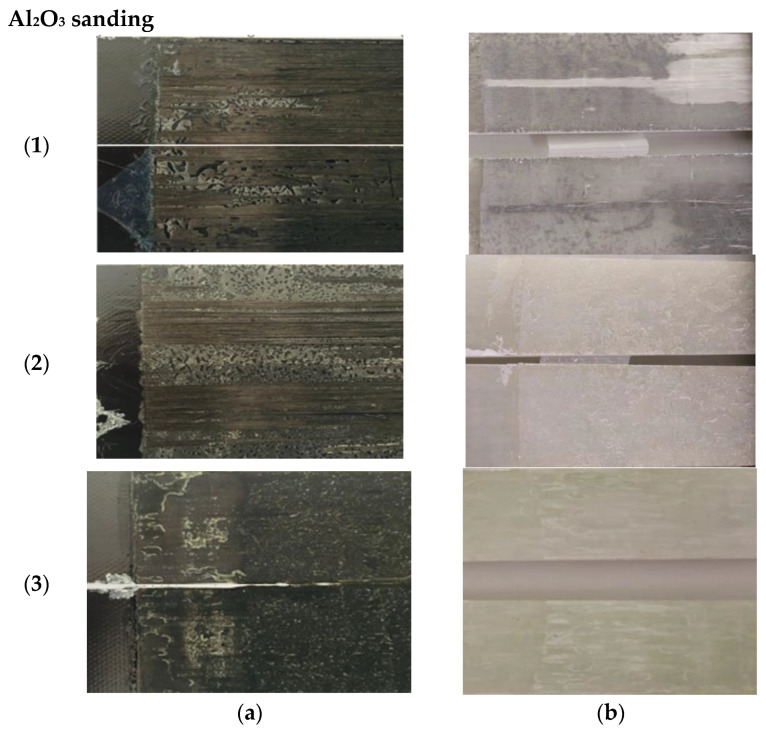
Mode I fracture surfaces of the adhesive joints: (**a**) Carbon and (**b**) Glass substrates. (**1**) Loctite® EA 9461™, (**2**) Araldite® 2015, (**3**) 3M™ DP8010NS. Surface preparation by sanding.

Figure 6 shows the different surface failure modes obtained as a function of the substrate type (carbon or glass) for each of the adhesives used, in this case with surface preparation by sanding.

Regarding the fracture typologies observed for the sanding surface treatment under Mode I loading, predominantly interlaminar failure within the composite substrate was identified for adhesives (1) Loctite^®^ EA 9461™ and (2) Araldite^®^ 2015, as indicated by fiber bridging. Consequently, the calculated G_IC_ values reflect the fracture resistance of the composite substrate rather than the intrinsic fracture toughness of the adhesive layer.

In contrast, for adhesive (3) 3M™ DP8010NS, a mixed failure mode was observed, with adhesive failure at crack initiation followed by cohesive failure during propagation.

For the glass-fiber substrate, adhesive (1) Loctite^®^ EA 9461™ exhibited mixed failure, predominantly cohesive. Both specimen faces were covered with adhesive, although small areas could be identified as adhesive failure since most of the adhesive remained on one side, leaving the other exposed (the white areas visible in the figure). For adhesive (2) Araldite^®^ 2015, the most common failure mode was intermediate between adhesive and cohesive: at the crack initiation, failure was generally cohesive, while adhesive failure areas increased as propagation advanced. For adhesive (3) 3M™ DP8010NS, the most recurrent failure was also intermediate between adhesive and cohesive, with fracture occurring such that part of the adhesive remained on one face and the other part on the opposite face.

**Figure 7:**
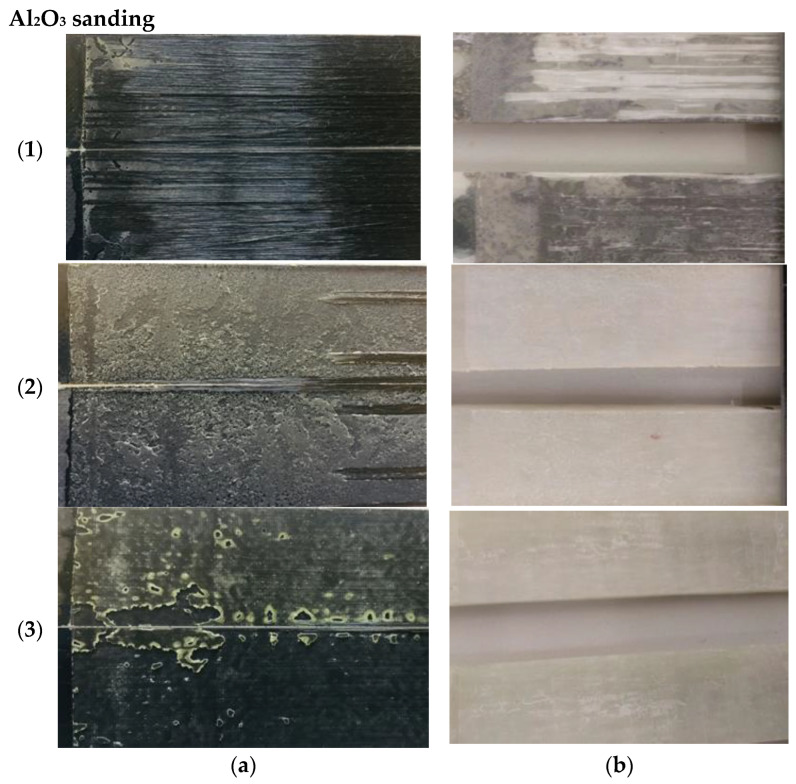
Fracture surfaces of the adhesive joints: (**a**) Carbon: (**1**) Loctite^®^ EA 9461™, (**2**) Araldite^®^ 2015, (**3**) 3M™ DP8010NS; (**b**) Glass: (**1**) Loctite^®^ EA 9461™, (**2**) Araldite^®^ 2015, (**3**) 3M™ DP8010NS. Surface preparation by grit blasting.

Figure 7 shows the most representative Mode I fracture surfaces for each of the different adhesives employed with the carbon and glass fiber substrates, when surface preparation prior to bonding was carried out by grit blasting.

For the carbon-based substrates, a clear difference was observed in the fracture surfaces between epoxy-based and acrylic-based adhesives. In the epoxy-based adhesives, predominantly cohesive or interfacial fracture was observed, often accompanied by significant fiber bridging. This suggests that crack propagation locally deviated into the composite substrate, particularly in regions where fiber bridging was present.

For the 3M™ DP8010NS adhesive, failure occurred mainly at the adhesive–substrate interface as shown in Figure 7(b3).

For the glass-based substrate, adhesive (1) Loctite^®^ EA 9461™ exhibited mixed failure, initially cohesive, with fiber bridging extending along the crack path and combined with adhesive failure. Adhesive (2) Araldite^®^ 2015 showed predominantly cohesive failure with minor regions of adhesive failure. In contrast, adhesive (3) 3M™ DP8010NS exhibited mainly adhesive failure, with no evidence of substrate damage.

**Figure 8:**
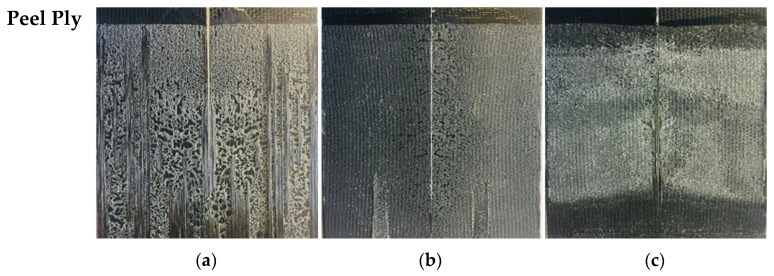
Fracture surfaces of the adhesive joints for the carbon-fiber composite: (**a**) Loctite^®^ EA 9461™, (**b**) Araldite^®^ 2015, (**c**) 3M™ DP8010NS. Surface preparation by Peel Ply.

Figure 8 shows the different surface failure modes obtained for each of the adhesives used when peel ply was employed as the surface preparation technique, only for the carbon-fiber-reinforced material.

With the peel ply technique, continuous cohesive failure was achieved throughout the crack propagation study. The fracture surfaces showed cohesive failure for all adhesives tested under Mode I loading. Overall, this technique produced the best fracture surfaces, as it resulted in continuous cohesive failure with the presence of fiber bridging, which, depending on the type of adhesive, occasionally increased fracture toughness.

Regarding the scatter in the results, relatively low variability was observed for the epoxy-based adhesives, as evidenced by the small standard deviation values represented by the error bars in Figure 5. In contrast, the acrylic-based adhesive 3M™ DP8010NS exhibited greater dispersion for the carbon-based substrate. This may indicate local failures in the laminate matrix and the presence of fiber bridging, which artificially modifies the fracture toughness of the joint.

It is important to emphasize that the three adhesives investigated exhibit inherently different mechanical properties and toughening mechanisms; therefore, a direct classification in terms of “high” or “low” performance would not be appropriate. Instead, the results should be interpreted in terms of sensitivity to surface preparation and response homogeneity.

Loctite^®^ EA 9461 and Araldite^®^ 2015 showed relatively homogeneous behavior across the different surface treatments, with moderate variations in G_IC_. In contrast, 3M™ DP8010NS exhibited a pronounced dependence on surface preparation, particularly under grit blasting, where a significant reduction in fracture toughness was observed compared to sanding and peel ply conditions.

These findings indicate that the global joint performance is governed not only by the intrinsic properties of each adhesive but also by the interaction between adhesive chemistry and the surface morphology generated by the preparation method. Therefore, surface treatment plays a critical role in optimizing fracture resistance, especially for adhesives that are more sensitive to interfacial conditions.

## 5. Conclusions

In this work, the influence of adhesive chemistry and surface preparation technique on the Mode I debonding behavior of adhesively bonded composite joints manufactured from CFRP and GFRP laminates with the same epoxy matrix was investigated.

The results demonstrate that surface preparation has a decisive influence on fracture performance. Although similar trends were observed for both substrates, the effectiveness of each treatment strongly depended on the adhesive type, highlighting the importance of adhesive–substrate compatibility.

Sanding provided consistently high and stable fracture toughness values for all adhesives and substrates investigated, making it a robust and reliable surface preparation technique. In contrast, grit blasting exhibited greater sensitivity to adhesive chemistry. For the acrylic-based adhesive 3M™ DP8010NS, blasting led to a pronounced reduction in G_IC_, which was associated with predominantly adhesive failure and reduced interfacial integrity.

Peel ply generated controlled resin-rich surfaces that promoted cohesive failure and stable crack propagation, leading to competitive fracture performance.

From a practical perspective, sanding is recommended as the most robust surface preparation method for both CFRP and GFRP substrates when using epoxy-based adhesives, as it provides consistently high and stable fracture toughness values. For the acrylic adhesive (3M™ DP8010NS), sanding and peel ply are preferable, whereas grit blasting should be avoided due to the significant reduction in interfacial performance. Peel ply can be considered an effective alternative when it can be integrated into the manufacturing process, as it promotes cohesive failure and stable crack propagation.

Overall, the results confirm that fracture toughness, which represents the energy required for crack propagation, is not solely governed by surface roughness but by the combined interaction between surface morphology, adhesive chemistry, and the resulting failure mechanism. Therefore, the optimal surface preparation method cannot be defined independently of the adhesive system employed.

## Figures and Tables

**Table 1:** Mechanical properties of the substrates used.

	Elastic Modulus ^a^		Tensile Strength ^a^		Shear Modulus ^b^	Shear Strength ^b^
Material	E_11_ (GPa)	E_22_ (GPa)	σ_11_ (MPa)	σ_22_ (MPa)	G_12_ (GPa)	τ_max_ (MPa)
MTC510-UD300-HS	122.0 CV = 8.5%	8.5 CV = 8.0%	1156.0 CV = 12.5%	28.0 CV = 11.8%	5.2 CV = 9.8%	37.0 CV = 2.0%
MTC510-UD300-Eglass	38.8 CV = 5.5%	8.4 CV = 6.0%	585.0 CV = 3.6%	41.9 CV = 0.8%	5.4 CV = 7.2%	34.3 CV = 8.3%

^a^ ASTM D 3039M-17R25 [47]. ^b^ ASTM D 3518M-18R25 [48].

**Table 2:** Basic properties of the adhesives used.

	Base	Viscosity [mPa·s]	Tensile Modulus [GPa]	Tensile Strength [MPa]	Shear Strength [MPa]
Loctite^®^ EA 9461^TM^	Epoxy	150,000 to 250,000	2.758	30.3	13.8
Araldite^®^ 2015	Epoxy	thixotropic	2.000	30.0	14.3
3M^TM^ DP8010NS	Acrylic	45,000	0.862	11.4	6.9

**Table 3:** Surface roughness values for the selected substrate surface preparation processes.

[µm]	As Received	Glass Bead Blasting, 5s	Al_2_O_3_ Blasting, 5s	Sanding, P220, Al_2_O_3_	Peel Ply	HNO_3_ Etching	HNO_3_ + HCl Etching
R_a_	2.21	3.09	3.20	3.14	9.34	2.25	3.68
R_z_	9.44	20.35	19.50	16.59	50.3	13.14	24.23
R_max_	10.91	21.23	23.98	18.88	59.99	18.45	26.08

## Data Availability

The original contributions presented in this study are included in the article. Further inquiries can be directed to the corresponding author.
